# Long non-coding RNA TTN-AS1 promotes breast cancer cell migration and invasion via sponging miR-140-5p

**DOI:** 10.3892/ol.2020.12018

**Published:** 2020-08-25

**Authors:** Jie Xue, Zhixia Zhang, Xiaona Li, Qingfang Ren, Qinghua Wang

Oncol Lett 19: 1255-1260, 2020; DOI: 10.3892/ol.2019.11222

Following the publication of this article, the authors contacted the Editor to request its retraction on account of having been unable to obtain the same results on performing further experiments. Subsequently, the Editorial office were also informed by an interested reader that data featured in Figs. 2 and 3 had also appeared in other non-related publications, and the office were able to confirm that these images were indeed the same as those featured in the other publications.

The Editor has agreed to the authors’ request, and therefore this article has been retracted from *Oncology Letters*. All the authors agree to this retraction. The authors apologize to the readership of the Journal for any inconvenience caused.

